# Trends in overall mortality among US veterans with primary myelofibrosis

**DOI:** 10.1186/s12885-022-10495-6

**Published:** 2023-01-14

**Authors:** Tsewang Tashi, Jingbo Yu, Shivani Pandya, Christopher Dieyi, Robyn Scherber, Shreekant Parasuraman

**Affiliations:** 1 Division of Hematology & Hematologic Malignancies, Huntsman Cancer Institute, University of Utah & SLC VAMC, Salt Lake City, UT 84112 USA; 2 Incyte Corporation, Wilmington, DE USA; 3 STATinMED LLC, Dallas, TX USA; 4 Baylor College of Medicine, Houston, TX USA; 5 UT Health San Antonio MD Anderson Cancer Center, San Antonio, TX USA

**Keywords:** Myelofibrosis, Ruxolitinib, Veterans, Mortality

## Abstract

**Background:**

Primary myelofibrosis [PMF] is a myeloproliferative neoplasm associated with reduced overall survival (OS). Management strategies for PMF have evolved over the last two decades, including approval of ruxolitinib as the first Janus kinase 1 (JAK1)/JAK2 inhibitor for patients with intermediate or high-risk myelofibrosis. This study assessed changes in mortality before and after ruxolitinib approval, independent of ruxolitinib treatment.

**Methods:**

This retrospective study investigated mortality trends among US veterans with PMF in 2 time periods, pre-ruxolitinib approval (01/01/2007–12/31/2010) and post-ruxolitinib approval (01/01/2015–09/30/2018). Deidentified patient-level data were extracted from US Veterans Health Administration (VHA) databases using PMF diagnosis codes; index was the first PMF diagnosis date. The analysis included adults with ≥2 PMF claims during the analysis periods who were continuously enrolled in the VHA plan 1 calendar year prior to and 6 months post-index and had ≥1 available International Prognostic Scoring System (IPSS) risk factor (available factors were age > 65, hemoglobin < 10 g/dL, and white blood cell count > 25 × 10^9^/L; each counted as one point). Patients with ≥1 MF diagnosis for 12 months before the index period were excluded. Ruxolitinib treatment was not a requirement to be included in the post-ruxolitinib approval cohort. Mortality rates and OS were estimated using the Kaplan-Meier approach; all-cause mortality hazard ratio was estimated using univariate Cox regression.

**Results:**

The pre- and post-ruxolitinib approval cohorts included 193 and 974 patients, respectively, of which 80 and 197 had ≥2 IPSS risk factors. Ruxolitinib use in the post-ruxolitinib cohort was 8.5% (83/974). At end of follow-up, median (95% CI) OS was significantly shorter in the pre-ruxolitinib cohort (1.7 [1.2–2.6] years vs not reached [3.4–not reached]; *P* < 0.001). Overall mortality rates for the pre- versus post-ruxolitinib approval cohorts were 79.8% versus 47.3%, respectively, and overall risk of death was 53% lower in the post-ruxolitinib period (hazard ratio, 0.47; 95% CI, 0.37–0.58; *P* < 0.001). Mortality rates were lower among patients with < 2 vs ≥2 IPSS risk factors.

**Conclusions:**

Although veterans with PMF have high overall mortality rates, and results in this population might not be generalizable to the overall population, there was a significant lowering of mortality rate in the post-ruxolitinib period.

## Background

Myelofibrosis (MF) is a myeloproliferative neoplasm characterized by bone marrow fibrosis, extramedullary hematopoiesis, and burdensome constitutional symptoms [1, 2]. MF can be either primary (PMF), arising de novo, or secondary to transformation from preceding polycythemia vera (PV) or essential thrombocythemia (ET), termed as post-PV or post-ET MF, respectively [2]. PMF is associated with reduced overall survival (OS) compared with the survival of healthy controls matched for age, sex, and calendar period [3–5]. Median OS in patients with PMF ranges from 2.3 to 11.3 years from diagnosis depending on the patients’ International Prognostic Scoring System (IPSS) risk score calculated at diagnosis [6]. The risk factors included in the calculation of the IPSS score are age (> 65 years), the presence of constitutional symptoms, hemoglobin (Hb) levels < 10 g/dL, white blood cell (WBC) count > 25 × 10^9^/L, and blasts ≥1%. Patients with PMF can be classified as low risk, intermediate-1, intermediate-2, or high risk if they have 0, 1, 2, or ≥ 3 risk factors, respectively [6].

Treatment for PMF was largely supportive in the past [7] before the emergence of Janus kinase 2 (JAK2) inhibitors, which substantially improved the overall outcomes in these patients, as demonstrated by spleen volume reduction, disease-related symptom improvement, and improved OS observed in clinical trials [8]. Ruxolitinib is an oral selective inhibitor for JAK1 and JAK2 that showed improved OS in patients with intermediate- or high-risk MF in the COMFORT-I and –II phase 3 trials [9–11]. These findings were further substantiated by a pooled analysis of the COMFORT-I and –II trials and the postmarketing phase 3 JUMP trial, which evaluated ruxolitinib in patients with intermediate-1, intermediate-2, or high-risk MF, including those with low platelet counts at baseline [12, 13]. Ruxolitinib was approved by the US Food and Drug Administration (FDA) in November 2011 [14], and as an inhibitor of JAK1 and JAK2 became the only JAK2 inhibitor that was FDA approved for MF until 2019, when a second JAK2 inhibitor, fedratinib (selective inhibitor of JAK2), was approved for patients with intermediate-2 or high-risk MF [15].

The introduction and availability of JAK2 inhibitors in PMF provides an opportunity to study the impact of the evolving treatment landscape on real-world patient outcomes, including OS. Data on the OS of veterans with PMF are limited and represent a unique opportunity to assess this impact, given the longitudinal follow-up of patients in the Veterans Health Administration (VHA) system. In this study, mortality trends among US veterans with PMF were examined in the last two decades: before and after the availability of ruxolitinib, independent of ruxolitinib treatment.

## Methods

### Study design and patients

**Fig. 1:**
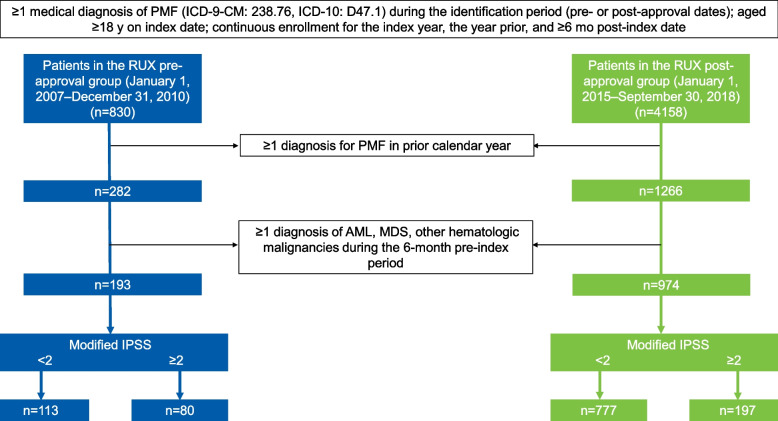
Patient Attrition. AML, acute myeloid leukemia; ICD, International Classification of Diseases; IPSS, International Prognostic Scoring System; MDS, myelodysplastic syndrome; PMF, primary myelofibrosis; RUX, ruxolitinib

This was a retrospective study using deidentified patient-level data obtained from the VHA. The VHA is the largest integrated health care system in the United States, providing care for approximately 9 million veteran and nonveteran enrollees across the country, and includes 153 medical centers and 882 ambulatory care and community-based outpatient clinics, among other resources. Both veterans and nonveterans were eligible for the study, although most patients (~ 90%) enrolled in the VHA are veterans [16]. Adult patients with PMF were identified using International Classification of Diseases (ICD) diagnosis codes relevant to PMF (ICD-9-CM: 238.76, ICD-10: D47.1); eligible patients had ≥2 claims of PMF during the respective analysis periods. Included in the analysis were patients from 2 VHA data periods (January 1, 2007–December 31, 2010 and January 1, 2015–September 30, 2018) reflecting the years before and after ruxolitinib approval (Fig. 1). These time periods were selected based on the availability of data limited to only before or after ruxolitinib in 2011; therefore, no data from 2011 to 2014 were available for inclusion. The index date was defined as the first PMF diagnosis in the identification periods. Patients with ≥1 diagnosis of acute myeloid leukemia, myelodysplastic syndrome, or other hematologic malignancies in the 6-month pre-index period and those with ≥1 MF diagnosis during the 12-month pre-index period were excluded from the study. Furthermore, patients were required to be continuously enrolled in the VHA system from 1 calendar year before to 6 months after the index date. Patients were followed up retrospectively per the study design, with follow-up period censored at earliest of death or end of data availability, whichever occurred first. The end of data availability was the end year for each group (2010 and 2018 for the pre- and post-ruxolitinib approval periods, respectively). Ruxolitinib treatment was not a requirement for patients to be included in the post-ruxolitinib approval cohort. Hb levels and WBC counts at diagnosis were based on results from the laboratory test closest to index within a 6-month period pre- and post-index. Charlson Comorbidity Index was calculated as described previously using ICD-9-CM codes [17]. A “modified” IPSS scoring system was used based on the 3 of 5 variables available from patients’ charts: age > 65 at index date, Hb levels < 10 g/dL at index, and WBC count > 25 × 10^9^/L at index. The presence of each criterion was counted as 1 point. Patients were categorized as having < 2 or ≥ 2 modified IPSS scores. Information on the other two variables (constitutional symptoms and percentage circulating blast cells) was not available in the database.

This retrospective study was performed in accordance with ethical principles that have their origin in the Declaration of Helsinki and conducted in adherence to the study Protocol. The study was exempt from institutional review board or ethics committee approval per Exemption 4 of the Office for Human Research Protections Regulations for the Protection of Human Subjects (45 CFR 46) as it used only deidentified claims data from the United States Department of Veterans Affairs. Deidentification and exemption from 45 CFR 46 was determined by a third-party consultant (dEpid/dt Consulting, Inc., Pleasant Ridge, MI). The data is licensed under an agreement with the VHA and not publicly available. Expert determination of statistical deidentification was in compliance with Section 164.514(b) of the Health Insurance Portability and Accountability Act (HIPAA) Privacy.

### Statistical analyses

Demographic and clinical characteristics were reported for all patients included in the study. All-cause 1-year, 2-year, 3-year, 5-year, and overall mortality rates, as well as OS from index until end of data availability, were estimated using the Kaplan-Meier approach. OS is defined as the time from diagnosis to death. The hazard ratio (HR) for all-cause mortality for the pre- and post-ruxolitinib cohorts was estimated using univariate Cox regression.

A subgroup analysis of survival was also performed stratified by modified IPSS score (< 2 vs ≥2). Modified IPSS was used in this analysis because IPSS is the recommended risk stratification tool for use at diagnosis compared with dynamic IPSS (DIPSS), which is recommended for use during the course of treatment [2].

## Results

### Baseline patient characteristics

The pre-ruxolitinib cohort included 193 patients with PMF, of which 80 (41.5%) had a modified IPSS risk score ≥ 2 at diagnosis, whereas in the post-ruxolitinib cohort, 197 (20.2%) out of 974 included patients had a modified IPSS risk score ≥ 2 (Table 1). In both the pre- and post-ruxolitinib cohorts, most patients were age ≥ 65 years at diagnosis (72.5 and 72.6%, respectively), male (99.0 and 95.0%), and White (59.1 and 72.9%). At PMF diagnosis, 47.7% of evaluable patients in the pre-ruxolitinib cohort had Hb < 10 g/dL, compared with 19.1% in the post-ruxolitinib cohort. Furthermore, 15.0% of evaluable patients in the pre-ruxolitinib cohort had a WBC count > 25 × 10^9^/L versus 9.1% in the post-ruxolitinib cohort. Finally, actual ruxolitinib use in the post-ruxolitinib cohort was only 8.5% (83/974).

**Table 1:** Patient baseline demographics and clinical characteristics

Characteristic	Overall Population		< 2 Modified IPSS Risk Factors		≥2 Modified IPSS Risk Factors	
	Pre-RUX Approval (***n*** = 193)	Post-RUX Approval (***n*** = 974)	Pre-RUX Approval (***n*** = 113)	Post-RUX Approval (***n*** = 777)	Pre-RUX Approval (***n*** = 80)	Post-RUX Approval (***n*** = 197)
Age, y, mean (SD)	71.2 (11.2)	69.3 (11.4)	67.1 (11.6)	67.9 (11.9)	77.1 (7.3)	74.8 (7.0)
≥ 65 y, n (%)	140 (72.5)	707 (72.6)	62 (54.9)	515 (66.3)	78 (97.5)	192 (97.5)
Male, n (%)	191 (99.0)	925 (95.0)	112 (99.1)	731 (94.1)	79 (98.8)	194 (98.5)
Race, n (%)						
White	114 (59.1)	710 (72.9)	69 (61.1)	569 (73.2)	45 (56.3)	141 (71.6)
Black	18 (9.3)	176 (18.1)	8 (7.1)	136 (17.5)	10 (12.5)	40 (20.3)
Other	61 (31.6)	28 (2.9)	36 (31.9)	20 (2.6)	25 (31.3)	8 (4.1)
Unknown	0	60 (6.2)	0	52 (6.7)	0	8 (4.1)
Charlson Comorbidity Index, mean (SD)	1.42 (1.99)	1.49 (2.04)	0.97 (1.64)	1.29 (1.81)	2.06 (2.25)	2.29 (2.63)
Hb measured, n (%)	193 (100)	908 (93.2)	113 (100)	714 (91.9)	80 (100)	194 (98.5)
Hb < 10 g/dL, n (%)^a^	92 (47.7)	173 (19.1)	21 (18.6)	22 (3.1)	71 (88.8)	151 (77.8)
WBC count, n (%)	193 (100)	923 (94.8)	113 (100)	736 (94.7)	80 (100)	197 (100)
WBC > 25 × 10^9^/L, n (%)^a^	29 (15.0)	84 (9.1)	23 (20.4)	6 (0.8)	23 (28.8)	78 (39.6)
Allogeneic HCT, n (%)	0	19 (2.0)	0	14 (1.8)	0	5 (2.5)
Splenomegaly^b^ (± 90 days of index)	0	54 (5.5)	0	30 (3.9)	0	24 (12.2)
RUX use, n (%)	–	83 (8.5)	–	39 (5.0)	–	44 (22.3)

*Hb* Hemoglobin, *ICD* International Classification of Diseases, *IPSS* International Prognostic Scoring System, *RUX* Ruxolitinib, *WBC* White blood cell

^a^Among patients with available measurements

^b^Based on ICD diagnosis code in the claims

### Survival estimates

**Fig. 2:**
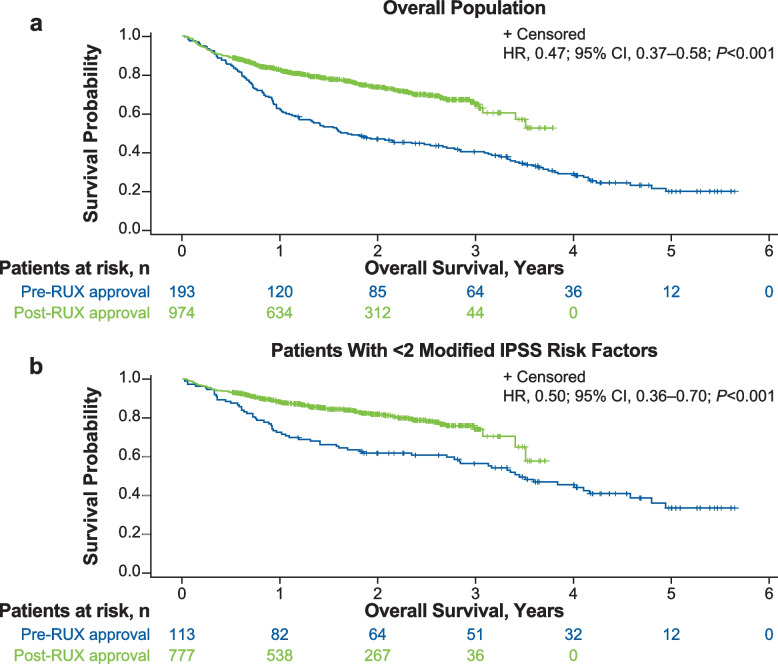
Overall Survival of Patients in Pre- and Post-Ruxolitinib Approval Groups in the **A** Overall Population, and **B** With < 2 Modified IPSS Risk Factors. HR, hazard ratio; IPSS, International Prognostic Scoring System; RUX, ruxolitinib

At the end of the follow-up period (median [IQR] follow-up, 17.0 [8.9–28.8] months), median (95% CI) OS was significantly shorter for the pre-ruxolitinib cohort (1.7 [1.2–2.6] years) versus the post-ruxolitinib cohort (not reached [3.4–not reached]; *P* < 0.001; Table 2 and Fig. 2A). The overall mortality rates for the pre- versus post-ruxolitinib cohorts were 79.8% vs 47.3%, respectively (Table 2). Furthermore, the 1-year mortality rate for the pre-ruxolitinib cohort was 37.8% compared with 17.6% for the post-ruxolitinib cohort. The 2-year mortality rates for the pre- and post-ruxolitinib cohorts were 53.0 and 26.3%, respectively. Overall risk of death was 53% lower in the post- versus pre-ruxolitinib cohort (HR, 0.47; 95% CI, 0.37–0.58; *P* < 0.001).

**Table 2:** Survival outcomes

	Overall Population		< 2 Modified IPSS Risk Factors		≥2 Modified IPSS Risk Factors	
	Pre-RUX Approval (***n*** = 193)	Post-RUX Approval (***n*** = 974)	Pre-RUX Approval (***n*** = 113)	Post-RUX Approval (***n*** = 777)	Pre-RUX Approval (***n*** = 80)	Post-RUX Approval (***n*** = 197)
**OS, median, y (95% CI)**	1.7 (1.2–2.6)	NR (3.4–NR)	3.4 (2.7–4.5)	NR (3.5–NR)	0.9 (0.8–1.3)	1.6 (1.1–2.0)
**Overall mortality rate,**^**a **^**n (%)**	137 (79.8)	242 (47.3)	63 (66.5)	134 (42.1)	74 (97.5)	108 (72.5)
**1-year mortality rate,**^**a **^**n (%)**	73 (37.8)	164 (17.6)	31 (27.4)	87 (11.8)	42 (52.5)	77 (40.0)
**2-year mortality rate,**^**a **^**n (%)**	102 (53.0)	216 (26.3)	43 (38.2)	116 (18.1)	59 (73.8)	100 (57.1)
**3-year mortality rate,**^**a **^**n (%)**	113 (59.5)	238 (35.1)	48 (43.5)	131 (25.8)	65 (81.6)	107 (67.9)
**5-year mortality rate,**^**a **^**n (%)**	137 (79.8)	–	63 (66.5)	–	–	–

*IPSS* International Prognostic Scoring System, *NR* Not reached, *OS* Overall survival, *RUX* Ruxolitinib

^a^Kaplan-Meier mortality rate estimate

Mortality rates were lower among patients with modified IPSS risk score < 2 compared with ≥2 (Table 2). Median (95% CI) OS for patients with a modified IPSS risk score < 2 was 3.4 (2.7–4.5) years for the pre-ruxolitinib cohort and not reached (3.5–not reached) for the post-ruxolitinib cohort (Table 2 and Fig. 2B). Risk of death was 50% lower in the post- versus pre-ruxolitinib cohort in patients with a modified IPSS risk score < 2 (HR, 0.50; 95% CI, 0.36–0.70; *P* < 0.001). In patients with a modified IPSS risk score ≥ 2, median (95% CI) OS for the pre-ruxolitinib cohort was 0.9 (0.8–1.3) years versus 1.6 (1.1–2.0) years for the post-ruxolitinib cohort (Table 2). The risk of death was lower in the post- versus pre-ruxolitinib cohort (HR, 0.73; 95% CI, 0.54–0.99; *P* = 0.04), although the results may be unreliable due to the low number of patients at risk after the first year.

## Discussion

In this retrospective analysis using data from the VHA database, patients with PMF had a high mortality rate in both the pre- and post-ruxolitinib periods (3-year mortality rate: 59.5 and 35.1%, respectively). When comparing similar follow-up periods, these mortality rates are consistent with previous reports in the literature. A retrospective study of 923 patients diagnosed with PMF between 1970 and 2009 had a relatively similar patient population, with a median (range) age of 65 years (14–92), 63% male patients, and 43% of patients having high IPSS risk disease. At a median follow-up of 34 months (~ 2.8 years), the mortality rate was 63% [18], which is closer to the mortality rate observed in the pre-ruxolitinib cohort of the current study. Another study involving patients with PMF from the Mayo Clinic database also had a majority of intermediate- or high-risk cases per DIPSS (89% intermediate-1/2 and high-risk) and found a mortality rate of 56% at a median follow-up of 3 years [19].

However, in this study, only 8.5% of patients in the post-ruxolitinib cohort received ruxolitinib treatment. This might be at least partially attributable to use of hydroxyurea as a cytoreductive therapy, especially in patients who had proliferative PMF, such as those with high WBC counts. Furthermore, per VHA criteria for use of ruxolitinib, the presence of multiple comorbidities or poor performance status (Eastern Cooperative Oncology Group [ECOG] > 3) in some veterans may have precluded them from using ruxolitinib [20]. Nevertheless, a 53% mortality risk reduction was observed compared to the pre-ruxolitinib cohort. There are multiple factors that may have influenced the lower mortality, but we believe the two key factors are the improved diagnostic strategy and better treatment options. Over the past 2 decades, there have been significant improvements in the diagnostic accuracy of the different clinical entities that encompass the spectrum of classical myeloproliferative neoplasms [21]. Besides the detection of driver mutations (ie, *JAK2*, *CALR*, and *MPL*), the use of next-generation sequencing in determining additional mutations in many myeloid gene panels is now widely available [22]. The World Health Organization has revised the criteria for diagnosis and classification of myeloproliferative neoplasms, including PMF, which now include the detection of driver mutations such as *CALR* and *MPL*, the increased importance of assessing morphologic findings in bone marrow biopsy to help with differential diagnosis of PMF, and the addition of a pre-PMF category [1]. It is very plausible that these improved and revised diagnostic criteria enable more accurate and earlier diagnosis and fewer misclassifications, which eventually result in better outcomes. Furthermore, it is reasonable to speculate that with improved symptom management, patients have better quality of life, which translates to better and healthier lifestyle, resulting in better overall outcomes.

After the arrival of JAK2 inhibitors, both the National Comprehensive Cancer Network® and European LeukemiaNet guidelines were updated to include JAK2 inhibitors as a first-line treatment option for MF in patients with intermediate-2 or high-risk disease [2, 23, 24], although ruxolitinib was the only FDA-approved JAK2 inhibitor during the study period of the current analysis. Both ruxolitinib and fedratinib have been shown to meaningfully improve patient outcomes. The 2 phase 3 COMFORT trials demonstrated that ruxolitinib significantly prolongs OS and reduces splenomegaly compared with placebo or best available therapy. Furthermore, ruxolitinib was associated with improved symptom burden and overall quality of life compared with best available therapy, based on patient-reported outcome data from COMFORT-I [10–12, 25, 26]. Real-world studies also suggest survival benefits for patients with MF treated with ruxolitinib in the past decade, although real-world data are limited [27–29]. One real-world study in particular analyzed data from patients with MF in the Medicare claims database (and therefore of older ages similar to the patient population in the current analysis), reporting improved survival in the post- versus pre-ruxolitinib approval era, with further survival benefit among patients exposed versus not exposed to ruxolitinib. The current analysis bolsters these findings, highlighting improved survival following ruxolitinib approval, at least in older patient populations [29]. Looking beyond ruxolitinib, fedratinib treatment was associated with reduced splenomegaly and a clinically meaningful improvement in MF symptoms and overall health-related quality of life compared to placebo, both in JAK inhibitor–naive patients and in patients who had previously received ruxolitinib [30–33]. Thus, the arrival of JAK2 inhibitors for PMF treatment contributes to better management of PMF and additional survival improvements.

Limitations of the current study are typical for retrospective database analyses, including potential misdiagnosis or miscoding in documentation (eg, identification of splenomegaly through diagnosis codes) and possibly incomplete records if patients sought care outside of VHA system. Even though the post-ruxolitinib cohort had better OS, the actual documented use of ruxolitinib in the post-approval cohort was low, reasons for which are not entirely clear. This may have affected the magnitude of outcomes and prevents any direct attribution of survival benefits to ruxolitinib use. Furthermore, the VHA population differs from the general population in various demographic and clinical characteristics; the vast majority of patients are older men and have a high rate of cardiovascular risk factors and other comorbidities such as hypertension, obesity, and diabetes [34]. There are also certain VHA-mandated exclusion criteria for receiving ruxolitinib, including that patients must have an ECOG performance status score ≤ 3 [20]. Thus, these results may not generalize to the broader patient population. In addition, 2 of the IPSS scoring system variables (constitutional symptoms and percentage of circulating blast cells) were not available in the database, which may have led to potential misclassifications (most likely underestimation of risk level) of patients as having a modified IPSS score of < 2 or ≥ 2, when in fact they may belong to a higher risk category. Also related to IPSS classification, there was a substantially lower proportion of patients with low Hb levels in the post-ruxolitinib cohort compared with the pre-ruxolitinib cohort. Although it is not possible to know the reasons behind this discrepancy, it is possible that overall improvements in patient symptom assessment and management contributed to this change. The current analyses were not adjusted for the potential use of other treatments besides ruxolitinib; however, ruxolitinib is the only pharmacological treatment that has been shown to impact OS in PMF [12]; thus, the effects of other treatments may have been minor.

## Conclusions

In summary, this retrospective analysis using data from the VHA database showed that patients with PMF had a high mortality rate both before and after ruxolitinib approval. A 53% lower risk of mortality was observed for patients diagnosed after ruxolitinib approval, possibly due to overall improvements in diagnosis and management of PMF. However, given that only a small portion of patients in the post-ruxolitinib group received ruxolitinib in this study, future analyses should examine mortality differences in patients who received ruxolitinib compared with those who did not, using data from real-world clinical practice.

## Data Availability

Access to individual patient-level data is not available for this study. Information on Incyte’s clinical trial data sharing policy and instructions for submitting clinical trial data requests are available at: https://www.incyte.com/Portals/0/Assets/Compliance%20and%20Transparency/clinical-trial-data-sharing.pdf?ver=2020-05-21-132838-960.

## Abbreviations

CI: Confidence interval
ET: Essential thrombocythemia
FDA: Food and Drug Administration
Hb: Hemoglobin
HR: Hazard ratio
ICD: International Classification of Diseases
DIPSS: Dynamic International Prognostic Scoring System
IPSS: International Prognostic Scoring System
IQR: Interquartile range
JAK: Janus kinase
MF: Myelofibrosis
OS: Overall survival
PMF: Primary myelofibrosis
PV: Polycythemia vera
VHA: Veterans Health Administration
WBC: White blood cell
